# Aging and Gaming: The Science and Promise of Technology-Based Leisure Activities and Interventions

**DOI:** 10.1093/geroni/igaa057.1835

**Published:** 2020-12-16

**Authors:** Chantal Kerssens

## Abstract

Much research has focused on technology to support older adults in basic and instrumental activities of daily living. Much less is known about technology supports of hobbies and leisure later in life. Physical, cognitive and social activities potentially delay the onset and progression of disease, including dementia. Older adults are interested in digital games, applications (apps) and social technologies, and will use technology provided their needs, preferences and goals are met. Moreover, video games can be designed to promote satisfying social experiences between players of differing capabilities. More work, however, is needed to understand older adults’ interactions and engagement with game-based interventions. This symposium presents cutting-edge research findings and design recommendations for technology-based leisure activities and interventions in older adults. Yow et al. present data from a large, touch-screen dual language intervention program with cognitive training tools aimed at slowing down the rate of cognitive decline in older adults with dementia. Boot et al. present longitudinal data from the Center for Research and Education on Aging and Technology Enhancement (CREATE) with a focus on leisure and videogames; Freed et al. present older adults’ attitudes and experiences with an exercise videogame (exergame). Lin et al. discuss the early effects of an exergame involving real-world physical activity on activity, social contact and stress levels in dementia caregivers. Kerssens et al. discuss the creation and testing of an adapted, accessible version of beloved board games for people with mild cognitive impairment (MCI) and a care partner without MCI. Technology and Aging Interest Group Sponsored Symposium.

